# A Study of a QCM Sensor Based on TiO_2_ Nanostructures for the Detection of NO_2_ and Explosives Vapours in Air

**DOI:** 10.3390/s150409563

**Published:** 2015-04-22

**Authors:** Marcin Procek, Agnieszka Stolarczyk, Tadeusz Pustelny, Erwin Maciak

**Affiliations:** 1 Department of Optoelectronics, Silesian University of Technology, 2 Akademicka St., 44-100 Gliwice, Poland; E-Mails: Tadeusz.Pustelny@polsl.pl (T.P.); Erwin.Maciak@polsl.pl (E.M.); 2 Department of Physical Chemistry and Technology of Polymers, Silesian University of Technology, 9 Strzody St., 44-100 Gliwice, Poland; E-Mail: Agnieszka.Stolarczyk@polsl.pl

**Keywords:** gas sensors, Quartz Crystal Microbalance (QCM), nanoparticles, titanium dioxide (TiO_2_), NO_2_ detection, nitroglycerine detection

## Abstract

The paper deals with investigations concerning the construction of sensors based on a quartz crystal microbalance (QCM) containing a TiO_2_ nanostructures sensor layer. A chemical method of synthesizing these nanostructures is presented. The prepared prototype of the QCM sensing system, as well as the results of tests for detecting low NO_2_ concentrations in an atmosphere of synthetic air have been described. The constructed NO_2_ sensors operate at room temperature, which is a great advantage, because resistance sensors based on wide gap semiconductors often require much higher operation temperatures, sometimes as high as 500 °C. The sensors constructed by the authors can be used, among other applications, in medical and chemical diagnostics, and also for the purpose of detecting explosive vapours. Reactions of the sensor to nitroglycerine vapours are presented as an example of its application. The influence of humidity on the operation of the sensor was studied.

## 1. Introduction

Systems for monitoring and detecting gases require the application of sensitive sensors, allowing one to detect and to determine low concentrations of single parts per million (ppm), and even parts per billion (ppb) in the air [1,2]. Gas sensors can also be used in medical diagnostics [3,4], in the chemical and food industry [5,6], and also in other fields [7,8,9]. Nitrogen oxides (NO_x_) constitute an important group of toxic gases, whose concentrations must be controlled due to their considerable detrimental effects. Nitro compounds are also components of the vapours of several explosive compounds, such as trinitrotoluene (TNT), hexogen (RDX) and nitroglycerine (glycerol nitrate—NG) [10]. 

An important group of sensors, effective in the detection and determination of concentrations of NO_x_ is based on wide band gap semiconductive metallic oxides such as TiO_2_, SnO_2_, WO_3_, In_2_O_3_, ZnO, Fe_2_O_3_ and combinations of these semiconductors [11,12,13,14,15,16,17,18,19]. Sensors based on these semiconductors may be applied in various types of gas sensors, among others in resistive sensors [20], optical sensors [21,22] and surface acoustic wave sensors [13]. Attempts have also been made to use TiO_2_ in systems for detecting gases (including reducing gases), such as NH_3_, CO, H_2_, H_2_S, alcohol vapours, moisture and others [23,24,25,26,27,28,29,30]. The paper deals with the possibility of using quartz crystal microbalance (QCM) acoustic mass sensors coated with TiO_2_ for detecting even minute concentrations of nitrogen oxides and nitroglycerine vapours in the air.

Gas sensors based on wide-band gap semiconductors often require high operation temperatures, generally within the range from 200 °C even up to 500 °C, as well as an activation of the sensors by means of ultraviolet (UV) radiation [31].

An adequate choice of the sorbent, which deposited on the transducer permits one to observe signal changes under the influence of adsorbed gas, is most important in the sensors of each mentioned type. The best-known and most widely applied sensors are thin-layer ones, which may be obtained by various methods, such as vacuum e-beam sputtering, magnetron sputtering or deposition from solutions, *etc.*

The sensibility of sensors should, however, be continuously improved and their time of response ought to be reduced, decreasing simultaneously the dimensions of the sensors and their energy consumption. This can be achieved by improving the morphology and topology of the surface of the gas-sorptive elements. In recent years nano- and microstructures, whose surface is incomparably larger than that of thin layers, have become very interesting in gas sensor techniques. Various methods of obtaining TiO_2_ nanostructures, for instance hydrothermal, sol-gel, chemical vapour deposition (CVD) methods, have been developed [32,33,34]. 

This paper focuses on the development of sensors based on a QCM containing sensor layers with nanostructures of TiO_2_. The method of synthesizing these nanostructures on the surface of the transducer is also presented. The elaborated prototype QCM system, as well as the results achieved during the measurements of small concentrations of NO_2_ and vapours of explosive materials in an atmosphere of synthetic air are discussed. The impact of humidity, hydrogen and ammonia on the operation of the sensors has been tested, too. 

## 2. Mechanisms of the Adsorption of NO_2_ on TiO_2_

At room temperature in the presence of oxygen and under anhydrous conditions NO_2_ is adsorbed on the surface of TiO_2_ according to the following reactions [35,36]:

Ti^4+^ + NO_2_ + O^2−^ ↔ Ti^3+^ + NO_3_^−^ (1)

TiO^3+^ + NO_2_ → TiO^4+^ + NO_2_^−^ (2)

Globally, this process occurs according to the reaction:

2NO_2_ + O^2−^ → NO_3_^−^ + NO_2_^−^ (3)

When a relatively high amount of analyte is adsorbed (about 3 μmol/m^2^ [36]) the result is a catalytic decomposition:

3NO_2_ + O^2−^ ↔ 2NO_3_^−^ + NO (4)

Under actual sensor operation conditions, a complete elimination of humidity is practically impossible. Commonly used commercial dehumidifiers allow one to decrease the relative humidity to a level of about 5% at room temperature. In the presence of humidity on the TiO_2_ surface a co-adsorption of water particles occurs, which leads to the formation of nitric acids [37]:

2NO_2_ + H_2_O → HNO_2_ + HNO_3_ (5)

Excessive levels of humidity can cause an accumulation of water on the surface of the absorber. As a result, a liquid film can emerge—hampering the evaporation of the adsorbed NO_2_ products. 

## 3. Experimental

### 3.1. Synthesis of the TiO_2_ Nanostructures

Nanofibres of TiO_2_ were obtained by means of the hydrothermal method [32]. For this purpose the following materials were used: anatase (Sigma Aldrich, Saint Louis, MO, USA), KOH and ethanol (POCH, Gliwice, Poland), and deionized water.

Into a Teflon vessel, anatase (0.5 g) and 10 M potassium hydroxide solution (20 mL) were introduced. The suspension was stirred for 10 min at room temperature and then placed into an autoclave. The reaction was carried out for 72 h at a temperature of 150 °C, the mixture being stirred the whole time. After its removal from the autoclave the sediment was decanted, repeatedly rinsed with deionized water, followed by 0.1 mol HCl solution until an acid reaction of the filtrate was achieved. The residual hydrochloric acid was rinsed with deionized water until a chemically inert pH was attained. 

**Figure 1:**
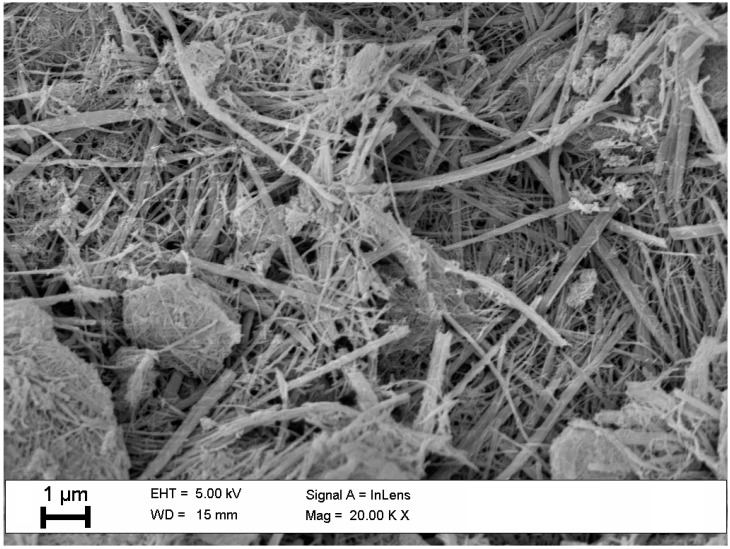
SEM image of TiO_2_ nanoparticles calcined at a temperature of 350 °C.

Then the obtained titanium acid (H_2_TiO_3_) was calcinated at a high temperature (higher than 200 °C). Nanostructures obtained in this way are discussed in our earlier work [20], where the temperatures of calcination were 350 °C and 450 °C. As the result of thermal calcination nanostructures in the form of powder were obtained. Examples of structures obtained by calcination at 350 °C are presented in the scanning electron microscopy (SEM) image in Figure 1.

It may be assumed that the nanostructures are morphologically similar and consist mainly of nanorods and nanotubes ranging in size from tenths of nanometers to a score of micrometers. TiO_2_ structures are rather inhomogeneous—the size and purity of the nanostructures differ depending on their position on the substrate.

### 3.2. Gravimetric QCM Transducers

**Figure 2:**
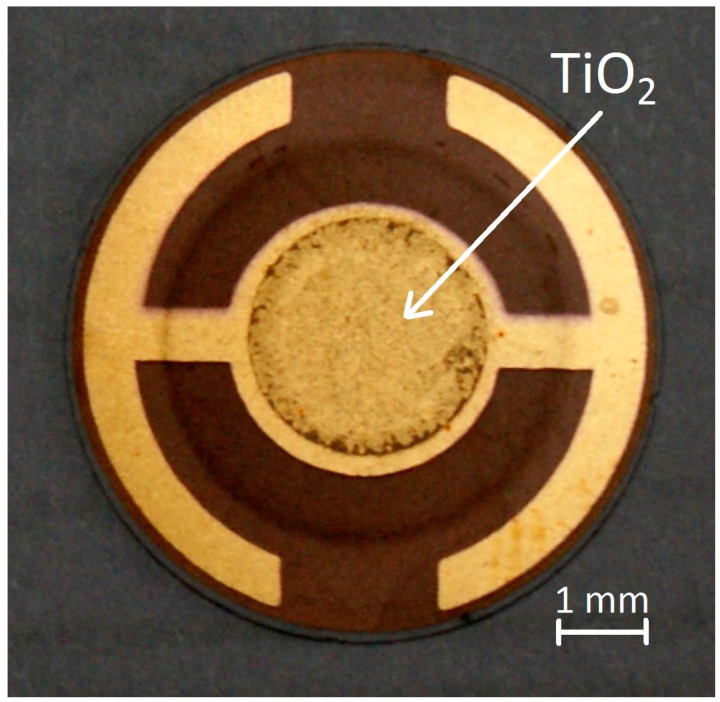
Completed QCM sensor with TiO_2_ nanostructures.

Applied gravimetric sensors are based on commercially available acoustoelectronic QCM transducers with a base frequency of 6 MHz. QCM transducers are made of AT-cut quartz plates (for this type of cut the frequency of oscillations depends weakly on the temperature). On both sides of the circular quartz crystal plate with a diameter of 14 mm, two gold electrodes were deposited. One of them covers completely the surface of the plate. The other one with a characteristic shape, deposited on the other side of the plate, is to be seen in Figure 2, where the TiO_2_ sensor layer was deposited. 

The idea of measuring the gas analytes is based on a change of the resonance frequency of vibrations of the quartz oscillator, affected by the mass accumulated on its surface. In order to detect volatile substances and to analyse the composition of the gaseous mixture, an adequate absorber has to be used. The sorbents have to adhere continuously to the transducer and be free of impurities in order to absorb the analyte. In our sensor the absorber is deposited merely on the central part of the transducer in the form of a circle with a diameter of 7 mm (Figure 2).

### 3.3. Deposition of TiO_2_ Nanostructures on the Transducer

The deposition of pulverized nanostructures on the transducer surface proved to be a rather non-trivial problem. The drop-coating method, applied to construct a resistive sensor [20], proved to be inadequate in the case of constructing the QCM sensor, because it did not ensure a proper adhesion of the nanostructures to the transducer. Several tests were carried out, attempting to improve the adhesion of the nanostructures to the transducer. Experiments of dispersing nanostructures were made in various liquids, *viz*. in ethyl alcohol, formaldehyde, acetone and toluene, without achieving an adequate adhesion. The performed investigations also comprised attempts to apply various kinds of bonds and glues like: nitrocellulose, polyvinylbutyral (PVB), ethyl cellulose, polyvinyl alcohol (PVA) and polyvinyl acetate (PVAc). 

In order to make the transducer operate adequately and to excite vibrations in the proper range of frequencies, its surface must be provided with an adequate amount of a sorbent whose mass is a fraction of a microgram. If the transducer is overloaded with material, the sensor fails to operate stably or ceases to vibrate in the required range of frequencies. The application of bonds is, therefore, an unfavorable solution, because it restricts the amount of the deposited absorber, or eliminates the application of commercial generators of vibrations for QCMs. Moreover, bonds partially destroy the absorbing surface of the nanostructures by clogging the micropores and hampering the penetration of the gas through the layer. Bonds may also react with the tested gaseous analytes, changing their chemical composition due to their reaction with reactive gases or due to the emission of vapours. 

In order to eliminate these negative phenomena, a new method of depositing TiO_2_ nanostructures on the sensors has been developed (without using any bonds). This method consists in the application of a hydrothermal procedure, in which titanium acid (H_2_TiO_2_) is calcined in the last stage directly on the surface of the transducer. Such a procedure ensures a better adhesion to the surface than the drop coating method, and also permits a reliable operation of the sensor by eliminating of the application of bonds. A patent has been filed for this method of obtaining this kind of sensor [38].

According to this investigation the method consists of the formation of a 0.5%–1.0% suspension of H_2_TiO_3_ in ethanol. On the QCM transducer a strictly adhesive circular mask was deposited. The suspension of H_2_TiO_3_ was dropped onto the mask and preheated at a temperature of 40 °C. The whole system was placed in an ultrasonic bath where it was heated to 40 °C and then left until the ethanol evaporated completely. Then the mask was removed and the sensor was calcined. The calcination process was started at room temperature and terminated at 220–250 °C. The process lasted about 2 to 4 h. The structures obtained as a result of calcinations are left in the furnace until they have cooled down. Higher calcination temperatures should be avoided, because they can lead to a mechanical destruction of the QCM transducer. A ready sensor made by means of the above-described method is shown in Figure 2.

Three independent runs of this process were carried out, where approximately ten functioning sensors were obtained. It is difficult to estimate the exact mass of the absorber as not all the material remained on the transducer after the calcination process (non-adhering material was removed), and the mass of the transducer is reduced during the calcination. The base frequency of the obtained sensors’ vibration can be taken as a measurement of the dispersion of the deposited amount of TiO_2_. This frequency differs from sensor to sensor by less than 0.1%. In all the cases, the adhesion of the material to the transducer was relatively good (the material did not peel off under a very strong jet of compressed air).

### 3.4. Measurement Chamber for the QCM Sensor

**Figure 3:**
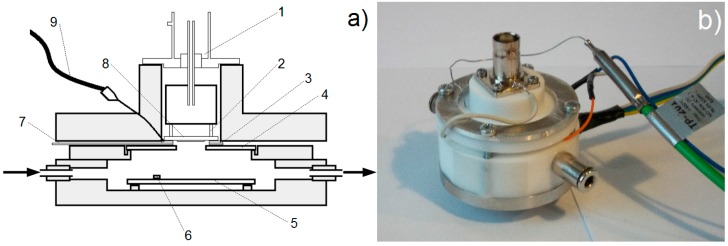
(**a**) Scheme of the measurement chamber for QCM sensor structure, where: 1—signal junction BNC, 2—measuring head with spring-actuated contacts, 3—conducting plate, 4—heating element with an orifice, 5—second heating element, 6—temperature gauge Pt-100, 7—conductive sheet—introduction of the mass, 8—sensor structure QCM with the deposited absorber, 9—temperature gauge—thermocouple; (**b**) photo of the QCM chamber.

In order to determine the sensitivity of the constructed QCM sensors for selected gases a special measurement chamber was designed and constructed. A complete measuring stand was also set up. The chamber is adapted to measure both gaseous analytes and vapours from solid samples. The measurement chamber was constructed of chemically inert materials such as PTFE and stainless steel. The applied materials are resistant to elevated temperature and do not emit gases even above 200 °C, being therefore resistant to aggressive gaseous environments. Construction details of the measuring chamber are shown in Figure 3.

The chamber was equipped with fast stainless steel gas couplers, which allow a fast supply of gas, as well as on the outlet from the chamber. Inside the chamber a heater, which is a thick-layered resistor deposited on an alumina substrate (Al_2_O_3_), as well as a Pt-100 temperature gauge are installed. The structure of the chamber allows controlled preheating of solid samples, e.g., explosive materials, in order to measure the composition of their vapours. The signal from the oscillator is fed to the QCM sensor by a measurement head provided with spring contacts. The head is directly connected with the measuring system by means of the BNC signal junction. The QCM sensor is embedded in an aperture in the cover, where the absorber is directed towards the orifice in such a way that the tested analytes are accessible to the sensor layer. The chamber has the shape of a cylinder with a diameter of 50 mm and a height of 50 mm (excluding the BNC juncture). All the electric contacts are passed through small orifices in the elements of the PTFE casing. The chamber is hermetic owing to the adequate openings cut in the PTFE elements.

### 3.5. Measurement Stand for QCM Sensors Tests

**Figure 4:**
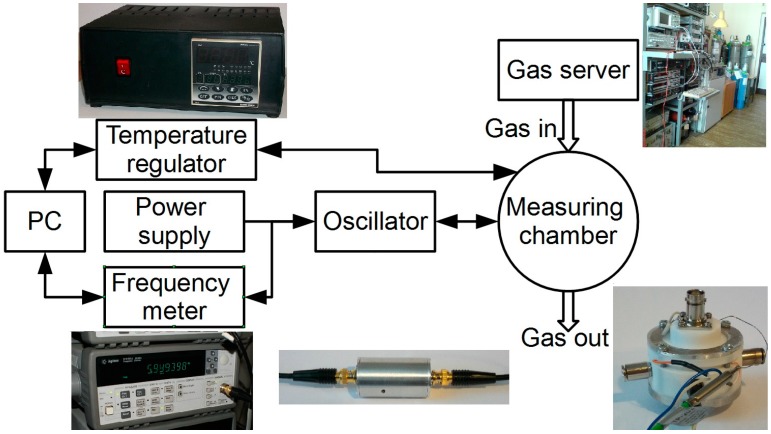
Block scheme of the measurement stand for QCM sensors.

The elements mentioned below were assembled to form the test stand, the scheme of which is shown in Figure 4. It contains a commercial oscillator (produced by Sycon Instruments, East Syracuse, NY, USA); the frequency is measured by a frequency meter (Agilent 53181A, Santa Clara, CA, USA); the temperature of solid samples is controlled by means of a temperature regulator (Shimaden FP93, Tokyo, Japan). The data acquisition is accomplished by means of a PC with the appropriate LabView software. The frequency meter is connected with the computer by means of a bus compatible with the GPIB standard. The test stand can be quickly plugged into a gas and vapour batching system. Gas mixtures were generated by a gas server with flow mass controllers, which ensures a constant flow of gas during the course of measurements. In all these experiments the applied carrier gas was synthetic air (20% O_2_ and 80% N_2_), so that the conditions were similar to natural ones. The humidity level was changed by the use of a humidifier with DI water. Sources of gases were cylinders with relevant calibration mixtures.

**Figure 5:**
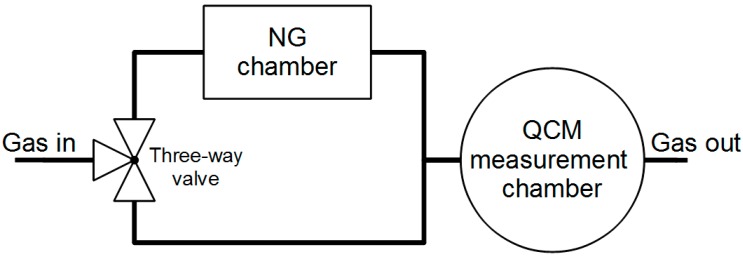
Scheme of the measurement stand for testing the response of the QCM sensor to nitroglycerine (NG) vapours.

In order to test the sensor responses to vapours of nitroglycerine the measurement system was modified as shown in Figure 5. In order to ensure a regeneration of the sensor by means of a clean carrier gas, a separate chamber with NG and a three-way valve was used. The valve permitted one to alternate the delivery of pure air and air containing NG vapours to the measurement chamber. 

The NG chamber was made of materials such as stainless steel and PTFE. The chamber provides the possibility of heating and stabilizing the temperature of the sample. A sample of 0.5 mg of nitroglycerine in diatomaceous earth was placed in the NG chamber, which was hermetically closed. Due to the continuous evaporation and degradation of NG its exact content in the sample was unknown and the composition of its vapours was not controlled. The aim of the experiment was to demonstrate the possibility of detecting the presence of NG (as an example of explosive material) at different temperatures.

The NG Chamber was flushed for 2 h by dry air (RH = 5.7%) with a flow rate of 1000 mL/min, in order to dry the sample and the elements of the measuring system. Then the sensor was heated up to 100 °C and the sensor chamber was flushed for 30 min with clean dry air in order to remove NG products and humidity. When the measuring system had cooled down, the appropriate measurements were carried out.

## 4. Results and Discussion

### 4.1. Characterization of the Sensor Structure

**Figure 6:**
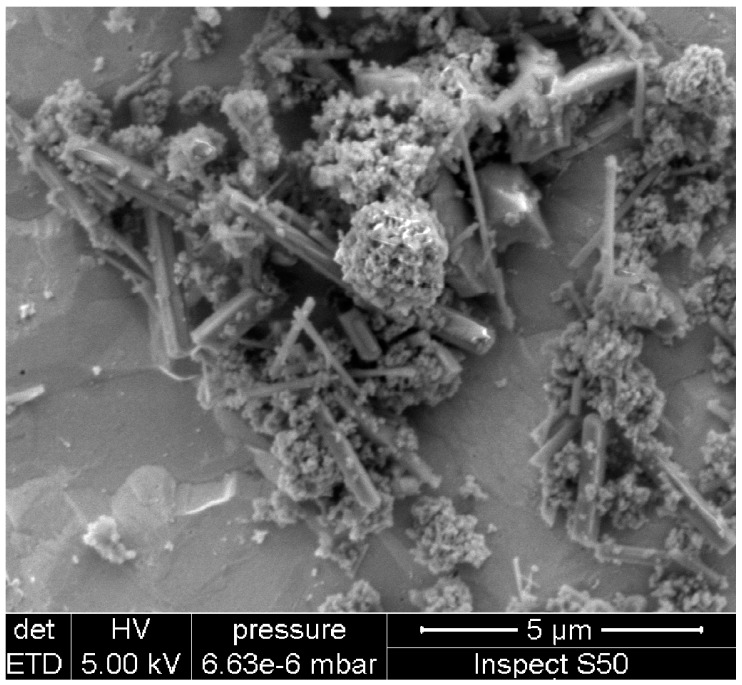
SEM image of the TiO_2_ sensor structure deposited on the QCM transducer.

The SEM image of the TiO_2_ sensor structure deposited on the QCM transducer is presented in Figure 6. The morphology of TiO_2_ structures deposited on the QCM transducer is slightly different from those which were calcined separately (Figure 1). These crystallites are in the form of nanowires with some admixture of other TiO_2_ nanocrystalline forms. The nanostructures are deposited flat on the transducer surface and adhere well to the substrate, because their crystallization started in the pores of a layer of gold. The obtained TiO_2_ sensor structure is porous, but it is difficult to estimate its roughness because of its heterogeneous morphology and lack of a homogeneous surface cover. For all the obtained sensors the morphology did not differ significantly, and the surface coverage was to a similar extent random.

**Figure 7:**
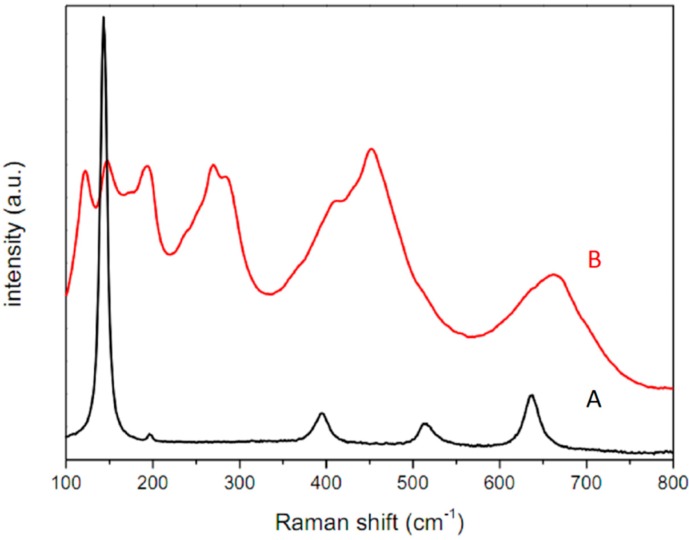
Raman spectra of: **A**—TiO_2_ after calcination on the QCM resonator and **B**—Titanium acid (H_2_TiO_3_) before calcination.

Raman spectroscopy was applied to characterize the TiO_2_ nanostructures—all the samples were similar, their peaks appearing at 143, 395, 514, and 634 cm^−1^, as shown in Figure 7A. This indicates a typical anatase TiO_2_ phase and reaction. The occurrence of the *in-situ* reaction on the surface of the sensor spectrum, characteristic of H_2_TiO_3_, is confirmed in Figure 7B. Thus, the Raman spectroscopy indicates that the material in the course of the calcination process completely crystallized into an anatase form.

### 4.2. Investigations of the QCM Sensors in an Atmosphere of NO_2_ in Synthetic Air

#### 4.2.1. Sensor Responses to a Wide Range of NO_2_ Concentrations in the Air

**Figure 8:**
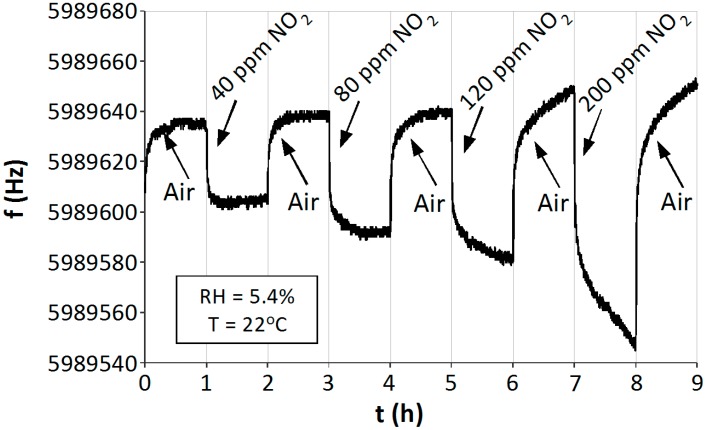
Response of the QCM sensor with a TiO_2_ absorber to NO_2_ in air at room temperature T = 22 °C and a humidity of the gas mixture RH = 5.4%.

**Figure 9:**
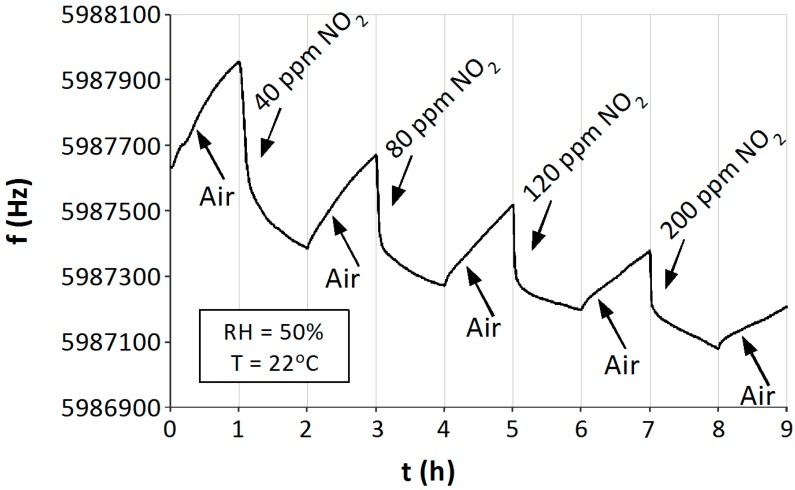
Response of the QCM sensor with a TiO_2_ absorber to NO_2_ in air at room temperature T = 22 °C and a humidity of the gas mixture RH = 50%.

The investigations were focused on the determination of the sensitivity of the sensors to NO_2_ and the effect of moisture on their operation. Special attention was paid to possible low temperatures of the sensor operation. The measurements were carried out at a continuous flow rate of the gas mixture amounting to 500 mL/min. The measurements were carried out at room temperature (22 °C) in conditions of dry (RH = 5.4%) and humid (RH = 50%) air (Figure 8 and Figure 9). The measurements were carried out in one-hour cycles, where pure air and air containing NO_2_ of a given concentration (within the range from 40 to 200 ppm) were alternatively introduced into the chamber. The concentration was increased in each successive cycle. Before each measurement the sensor was first exposed to NO_2_ at the concentration level of 20 ppm in order to stabilize its operation. Since the first exposure is unreliable, it is not shown in the diagrams, but the desorption can be observed (Figure 8 and Figure 9).

Under dry conditions of operation the QCM sensor signal was stable and cycles of one hour allowed a complete saturation of the absorber and its complete desorption (Figure 8). Gas molecules adhering to TiO_2_ increased the mass of the absorber, decreasing the frequency of QCM oscillations. When pure air was flowing through the measurement chamber, NO_2_ was desorbed from the sensor, and so the frequency of oscillations increased in the “direction” of the basic frequency. Changes in the frequency of the sensor, caused by the adsorption of NO_2_, increased with its concentration, thus the sensor becomes a scaled one. 

The operation of the sensor in humid conditions differed from that in dry conditions, as may be concluded from Figure 9. At the first exposure to NO_2_, an accumulation of the products of H_2_O and NO_2_ particles in the absorber can be already observed. Probably, the accumulation of water on the absorber causes in it a dissolution of NO_2_ and the formation of nitric acids (Equation (5)), the result of which is a high decrease of the frequency. It has been noticed that the desorption of H_2_O, NO_2_ and their products from the absorber based on TiO_2_ nanostructures for humid air is very weak in comparison with its sorption. This causes an occlusion of the sensor, which results in a frequency drift (Figure 9). The more the concentration of NO_2_ increases, the more the responses of the sensor decrease, so that the sensor characteristics tend towards lower operation frequencies. This proves an accumulation of products of NO_2_ and H_2_O reactions on the sensor structure. The frequency attained its basic value only after a long exposure to a flow of dry air or after the sensor has been heated up to more than 100 °C.

#### 4.2.2. Sensor Response to Low Concentrations of NO_2_

Another constructed QCM sensor was tested concerning the effect of low concentrations of NO_2_ in a dry atmosphere. The measurements consisted in alternatively introducing into the measurement chamber pure air and air containing NO_2_ in cycles of 30 min. The concentration of NO_2_ varied within the range from 1 ppm to 100 ppm, the relative humidity was RH = 5.7%. The temperature of the sensor was kept stable at room temperature (T = 23 °C). The flow rate of the gas during the measurements amounted to 500 mL/min. 

The results of the study are presented in the form of a calibration curve, where the response of the sensor is calculated as the difference of the frequency in the carrier gas and the frequency in the presence of the tested gas at a given concentration:

(6) $$\text{Δ}f=\left|f_{air}-f_{gas}\right|$$

**Figure 10:**
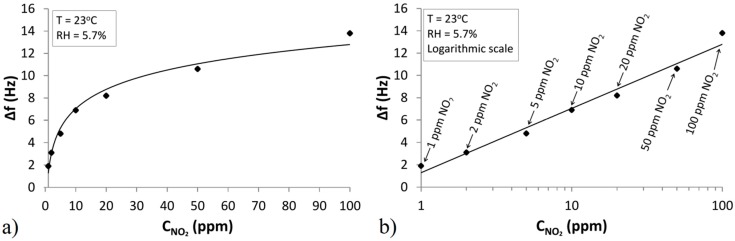
Sensor response to low concentrations of NO_2_, from 1 ppm to 100 ppm at room temperature T = 23 °C, in dry air RH = 5.7% (**a**) in linear scale; (**b**) in semi-logarithmic scale.

The calibration curve is shown in Figure 10, complying with the logarithmic relationship between the concentration and sensor response (Figure 10a). The semi-logarithmic scale was also used (Figure 10b). This characteristic shows that the sensor responds to low concentrations of NO_2_ starting around 1 ppm. 

The authors noticed that in the case of measurements of low concentrations of NO_2_ the desorption process is slower than in the case of higher concentrations. The differences in the rate of desorption are possibly caused by the presence of different products adsorbed on TiO_2_. These products result from the subsequent catalytic process, which is described by Reaction (4). Sensor readings at low concentrations of the analyte are therefore the sum of the quantity of the formed active Complexes (2) and the adsorbed final product NO_3_^−^ (3). It can be concluded that at low concentrations there is no accumulation of an appropriate amount of NO_2_^−^ for Reaction (4), thus the maximum concentration of the intermediate product is not achieved. At low concentrations the main product adsorbed on the sensor forms an active complex, which is the intermediate product of the subsequent reaction. At higher concentrations of NO_2_ Reaction (4) occurs quickly where one of the products is NO, which exhibits a low affinity to TiO_2_ at room temperature [36]. As a result, this part of the adsorbed analyte is removed from the sorbent. At the same time in the TiO_2_ structure the active centre recovers and the ratio of the adsorbed particles of NO_2_^−^ and NO_3_^−^ is changed. In the presence of water and a catalyst (TiO_2_) both these particles are subject to hydrolysis resulting in the formation of the appropriate nitric acids (H_2_NO_2_ and H_2_NO_3_) according to Reaction (5). The kinetics of the formation of both these acids will be different, and their release from the surface of TiO_2_ will occur at different rates. Therefore, at higher concentrations of NO_2_ the desorption process is faster. 

### 4.3. Sensor Response to Changes of Humidity

**Figure 11:**
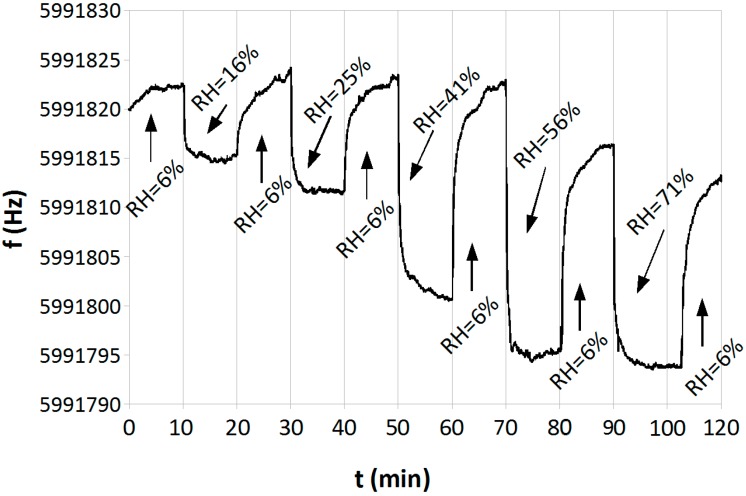
The influence of humidity on the operation of the QCM sensor at room temperature T = 23 °C, in a 500 mL/min gas flow.

The sensitivity of the sensor to changes of the humidity in the gas mixture was tested. The measurements were carried out at a constant flow rate of the gas, amounting to 500 mL/min. The relative humidity of the air varied from about 6% to about 70%. In each measurement cycle dry air (RH = 6%) and humid air were supplied alternately. The humidity was increased during each consecutive cycle. The temperature of the sensor was kept stable at RT = 23 °C. The results of this experiment are presented in Figure 11. It is evident that the sensor is very sensitive to changes of the relative humidity. When the RH is below 55%, changes in the frequency of the QCM sensor are proportional to changes of humidity, and in the course of desorption the sensor regains its basic frequency. Above RH > 55%, the sensor is saturated and does not react proportionally to the humidity. At a high level of humidity the desorption is retarded, and after the desorption it does not regain its basic frequency. This proves that a film of water is formed on the surface of the absorber in humid conditions.

When the moisture and NO_2_ affects the sensor simultaneously, the response of the measurement system is the sum of masses of the two analytes adsorbed in the sensor. As a result of the reaction of nitric oxide with water, in the presence of a catalyst (TiO_2_), low volatility nitric acids are formed (Reaction (5)), which are an additional mass on the sensor surface. As a result, during the operation of the sensor at a high level of humidity the system delivers a false positive response, namely an increase of the sensor response (Figure 9). Hence, it is to be concluded that the QCM sensor with a TiO_2_ sorbent requires a stable and possibly low humidity level of the investigated gas mixture, which can be ensured by using a proper gas dehumidifier (for example silica gel). Otherwise it does not operate correctly.

### 4.4. Sensor Response to Other Gases

The impact of hydrogen (in the range of 1% to 4%) and ammonia (in the range of 20 to 400 ppm) on the operation of the QCM sensor has been measured. The experiment was conducted at room temperature (RT = 23 °C) with a gas flow of 500 mL/min in dry air (RH = 5.7%). For both tested gases, the sensor showed no response exceeding the noise level (Table 1). This is due to the small absorption affinity of both gases to TiO_2_ at room temperature. This proves that the constructed sensor responds to NO_2_, but does not respond to H_2_ and NH_3_. The constructed sensor thus shows a selectivity to NO_2_ in presence of the mentioned gases. 

**Table 1:** Summary of sensor responses to different concentrations of tested gases. T = 23 °C, RH = 5.7%, carrier gas: air, gas flow: 500 mL/min for gases and 100 mL/min for NG vapours.

NO_2_	Concentration	1 ppm	2 ppm	5 ppm	10 ppm	20 ppm	50 ppm	100 ppm
	Response Δf, Hz	2	3	5	7	8	11	14
H_2_	Concentration	1%	2%	3%	4%	-	-	-
	Response Δf, Hz	0	0	0	0	-	-	-
NH_3_	Concentration	20 ppm	100 ppm	200 ppm	400 ppm	-	-	-
	Response Δf, Hz	0	0	0	0	-	-	-
RH	Concentration	16%	25%	41%	56%	71%	-	-
	Response Δf, Hz	8	12	22	27	29	-	-
NG	Sample temperature	23 °C	30 °C	35 °C	40 °C	-	-	-
	Response Δf, Hz	1.5	1.2	17.5	8.5	-	-	-

### 4.5. Detection of the Vapours of Explosive Materials

The sensor’s response to small concentrations of NO_2_ leads to the conclusion that the detection of explosives based on the detection of their vapours is possible. Materials, such as nitroglycerine (NG), trinitrotoluene (TNT) and RDX, which are hydrocarbons with nitro groups, undergo a slow degradation [12]. During this degradation nitrogen oxides such as NO and NO_2_ are released in small concentrations. Therefore, it was decided to test the response of the discussed sensor, based on TiO_2_ nanostructures, to the vapours of explosives. Nitroglycerine in diatomaceous earth was used as an example of explosive material. 

The measurements were carried out at room temperature (RT = 23 °C) with a continuous flow rate of gas amounting to 100 mL/min. During the measurement a three-way valve (Figure 5) was switched, so that the carrier gas alternatively flowed directly to the QCM measuring chamber or through the NG chamber. A single measurement cycle consisted of a flow of pure air through the QCM measuring chamber for 5 min and air with NG vapours for another 5 min, respectively. 

**Figure 12:**
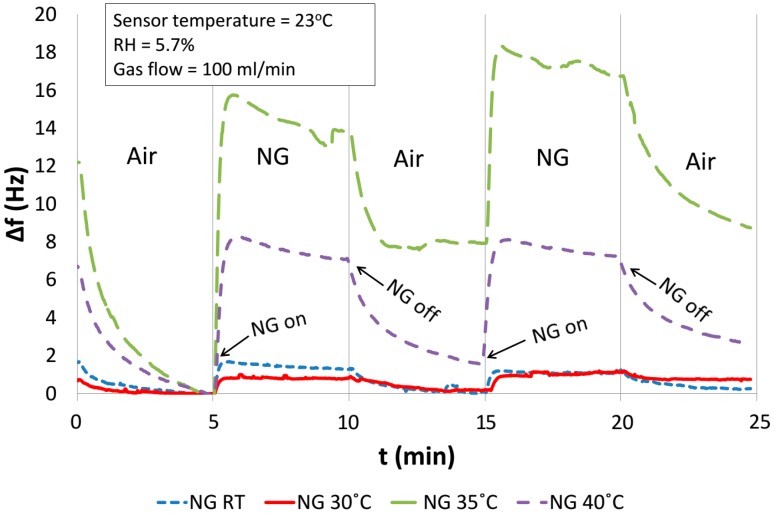
Response to nitroglycerine vapours in dry air (RH = 5.7%), sensor was at room-temperature (RT = 23 °C) and NG chamber temperature of: RT, 30 °C, 35 °C and 40 °C.

A single measurement comprised two such cycles and was completed by flushing with clean air. Four such measurements were made one after another, each at a different temperature of the sample in the NG chamber: RT, 30 °C, 35 °C and 40 °C, respectively (Figure 12). The temperature of the sensor was always kept at RT. It ought to be stressed that in each measurement the QCM sensor with TiO_2_ nanostructures responded to NG vapours. At room temperature and at 30 °C, the changes of the QCM frequency are comparably small and amount to about 1 Hz (the response is weaker than that of 1 ppm of NO_2_). After heating the NG up to 35 °C, much higher sensor frequency changes, up to 18 Hz, were observed. This considerable frequency change was a result of the accumulation of NG vapour during the heating of the chamber. Besides the first cycles, the results at temperatures of 35 °C and 40 °C showed similar sensor reactions to the effect of NG vapours. It must be noted that between the NG temperature of 30 °C and 35 °C, the amount of gaseous products of the degradation of the NG increased significantly, which leads to higher responses of the sensor at higher NG temperatures (Figure 12). The response time of the sensor to the presence of NG vapours is very short, and the maximum of the sensor response is achieved after approximately 1 min.

It can be seen that below 35 °C the response values of the sensor to NG vapours are similar to the response values in the case of small NO_2_ concentrations. The dynamics of frequency changes between the reactions to NG vapours and to humidity changes are different. An essential impact on the dynamic responses of NG vapours is caused by changes of their concentrations in time.

As pointed out earlier, before every single measurement cycle in the NG chamber the gaseous products of nitroglycerine had been accumulated and during the measurement they were diluted. Thus, the concentration of the vapours decreased during the measurement. This is reflected in the shape of the characteristics of the sensor’s frequency, where after a sharp frequency drop a slow growth is observed (Figure 12). This is why the desorption of the diluted analyte from the sensor is faster than in the case of NO_2_ where the concentration was kept at a constant level. 

The results obtained for NG prove that the sensor can detect the presence of nitroglycerine in dry air. Therefore, after appropriate drying of the gas sample, it is possible to detect the presence of nitro-explosive products in the air at room temperature. In order to summarize the investigations, sensor responses for all gases studied in this paper were calculated according to Equation (6) and summarized in Table 1.

## 5. Conclusions

The elaborated system, based on a QCM resonator combined with a sorbent deposited in the form of TiO_2_ nanostructures, is characterized by a relatively high and explicit sensitivity to NO_2_ in air at room temperature. This sensor requires however, a low and constant level of humidity in the range of 5% to 6% RH. This system permits to detect concentrations of NO_2_ as low as 1 ppm. 

A considerable advantage of the proposed sensor is the possibility to develop such a technology which would permit one to apply TiO_2_ nanostructures without the necessity of using bonds or glues in order to deposit them on the surface of QCM. Thanks to this, gaseous analytes can interact effectively with clean TiO_2_ structures, which adhere properly to the transducers. It is to be stressed, that the technology of constructing such sensors is relatively inexpensive and does not require more advanced technological installations. 

An essential property of such a solution is its operation at room temperature in the air, *i.e.*, in conditions similar to natural ones. The possibility of operating at room temperature and detecting a low concentration (about 1 ppm) of NO_2_ in the air, suggests its promising application in the near future. The sensor can be applied in chemistry, medicine, industry, biomedical engineering, as well as for detecting vapours of explosive materials, as experiments with NG prove that this sensor can detect nitro-containing explosives in air at room temperature. 

The performed investigations have proved that while measuring the presence of NO_2_ in the air the problem of the variability of humidity in the air must inevitably be solved. Moisture must be restricted in the analysed gaseous environment by absorption (by drying the air sample by dehumidifier). Our measurements have shown that in order to determine low concentrations of NO_2_ and NO_x_ in the air accurately, the humidity of air ought to be controlled and kept at a low level (for example RH = 6%). It has been noticed that in the case of higher humidity values a film of water is formed on the sorbent the. It causes an accumulation of the analyte and significantly impedes its desorption. 
